# Comprehensive Study of Scientific Evidence and Potential Risk of Herbal Medicine Use for Body Weight Reduction in North West Saudi Arabia

**DOI:** 10.7759/cureus.10903

**Published:** 2020-10-11

**Authors:** Fuad F Alfaifi, Abdulelah M Alghamdi, Bassam Al-omarani, Eyad H Dawaji, Hani Aljabry, Mohammed M Al-Gayyar

**Affiliations:** 1 PharmD Program, Faculty of Pharmacy, University of Tabuk, Tabuk, SAU; 2 Pharmaceutical Chemistry, Faculty of Pharmacy, University of Tabuk, Tabuk, SAU; 3 Biochemistry, Faculty of Pharmacy, Mansoura University, Mansoura, EGY

**Keywords:** herbal medicine, home remedies, north west saudi arabia, weight loss

## Abstract

Herbal and home remedies are widely used worldwide especially those for weight loss. The prevalence of overweight and obesity in Saudi Arabia has highly elevated during the past decades. People in Saudi Arabia preferred the usage of remedies more than standard medicine. Therefore, we conducted this study to assess the use of weight reduction herbal and home remedies in Tabuk, North West Saudi Arabia. Data about weight-loss herbal remedies were collected from major local herbalist shops. In addition, inherited weight-loss herbal and household remedies were collected. Data collected included composition, method of preparation, method of use, and any reported side effects. Some of these remedies were used either alone or in combination. Majority of these ingredients were boiled in water to be drunk or directly added to food. Most of these mixtures were taken before food to reduce appetite. Most of these herbs were recorded to produce side effects. Mainly people suffer from gastric symptoms and headache. Many natural herbal and home remedies are still in use in Tabuk for weight reduction. Many of these remedies lack important scientific evidence for their usage, effectiveness, or safety.

## Introduction

Overweight and obesity are well-known risk factors for development of many diseases such as type 2 diabetes mellitus, osteoarthritis, cardiovascular disease, fatty liver, obesity-related cancers, and mortality. It stands behind the deaths of at least 2.6 million people every year [1]. In addition, abdominal adiposity especially in the visceral tissue is strongly associated with body weight gain in adulthood and highly related to insulin resistance and risk of type 2 diabetes mellitus [2]. The World Health Organization (WHO) reported obesity as a global health threat. The problem has tripled since 1975. WHO reported that about 1.9 billion adults above 18 years were overweight in 2016. In addition, more than 40 million children under five years of age are overweight worldwide [3]. Therefore, obese children are now subjected to increased risk of health complications that were previously limited to adults, for example, type 2 diabetes mellitus and hypertension. It has been estimated that obese 10-year-old children range between 12,660$ and 19,630$ of incremental direct medical costs resulting in a potential of lifetime economic load of about 17 billion dollars [4].

The prevalence of overweight and obesity in Saudi Arabia has highly elevated during past six decades. A study at Riyadh, capital city of Saudi Arabia, revealed about 82% of participants were overweight or obese [5], while the prevalence of obesity in Jeddah, the second biggest city in Saudi Arabia, was 35% [6]. In addition, the prevalence of overweight and obesity in northwest region of Saudi Arabia was 69.9% [7]. Similar results were obtained from different studies covering different areas of Saudi Arabia. These major changes could be attributed to lifestyle transformation from traditional Saudi lifestyle into Western lifestyle, which are characterized by sedentary lifestyle and widespread of fast foods.

Several approaches have been made in the battle against obesity, such as clinical, dietary, and pharmacological approaches. Clinical approaches include surgery, lifestyle changes, and physical activity. Dietary approaches contain caloric restriction and weight-loss diet. Pharmacological approaches comprise several weight-loss drugs. However, the pharmacological approach utilizes many nonprescription herbals for weight loss without knowledge of their safety or efficacy [8].

People in developing and developed countries usually use drugs from natural sources. The type and use of herbal and home medicine vary according to the differences in cultures and availability of these materials. In order to maintain good health or treating diseases, people in Saudi Arabia preferred usage of complementary and alternative medicine either with or in replacement of standard medicine. Medical plants still constitute a major part of Saudis’ minds in the field of health care and represented a major source of medicine. However, we conducted this study to collect data about home remedies and herbal remedies that are used in weight loss either alone or in mixture in North West Saudi Arabia. In addition, we tried to evaluate the supporting data about their use.

## Materials and methods

Study design

This is a retrospective study that depends on collecting data about herbal and home remedies, which are used in weight loss. The study was conducted in the period between June and November 2019. The data was collected in Tabuk city located in the North West Saudi Arabia.

Data collection of herbal and home remedies

Data was collected about weight-loss herbal remedies that were either used alone or in combination with other herbs. The research team gathered all required information from the major local herbalist shops (20 shops) and traditional healers (10 places).

In addition, data was collected about the home remedies used for weight loss. The research team collected data about the inherited weight-loss remedies. These remedies are passed from one generation to another and used by many people in the area.

Meetings were conducted with senior residents in the area for more details about the herbal and home remedies used in Tabuk. Finally, composition, method of preparation, method of use, and any reported side effects were reported.

Evidence of efficacy of herbal and home remedies

A search of the literature was conducted to investigate all available evidence of usage, efficacy, and safety of active ingredients that are frequently used in weight reduction in Tabuk area. Literature citing the used ingredients were obtained from MEDLINE. The criteria depended on searching the name of the herbal or home remedy in the title or abstract using the limitation [Title/Abstract] in the search box of MEDLINE.

The research studies on English language were downloaded. The used studies were selected based on their scientific quality, especially randomized clinical trials when available, and originality. In addition, review studies on humans were preferred. Articles that met this criteria were 21, and they are used in the discussion.

## Results

Herbal medicine used for weight reduction

After collecting information about herbal medicines used in fighting obesity in Tabuk area, we found that some of these herbs were used either alone or in combination. As shown in Table 1, there were 12 herbs used alone for weight reduction. All of these single herbs were taken by mouth for systemic effect, and none of them were topically applied. Six herbs (50%) were boiled and drunk; another six (50%) were directly added to food. They were used to reduce appetite by taking before food (41.67%) or with food (50%). Out of these herbs, 11 (91.67%) were recorded to have side effects. Mainly people suffer from gastric symptoms such as heartburn, vomiting, and diarrhea, while few of them suffered from headache, dizziness, and hypoglycemia.

**Table 1:** Single herbs used in weight loss.

S. No	Common name	Scientific name	Major active constituents	Method of use	Reported side effects
1	Ginger	Zingiber officinale	Gingerol	Powder added to food. Boil with water and drink on empty stomach.	Cardiac arrhythmias, heartburn diarrhea
2	Green coffee beans and husks	Coffea arabica	Chlorogenic acid	Boil with water and drink.	Insomnia and nervousness, nausea and vomiting, tachycardia
3	Green tea	Camellia sinensis	Epigallocatechin, epicatechin, epigallocatechingallate, epicatecatechingallate	Boil with water and drink.	Headache and nervousness, heartburn, vomiting, dizziness
4	Beans	Phaseolus vulgaris	Phenolic compounds	Cook in water and eat.	Stomach upset
5	Hibiscus flowers	Hibiscus rosa-sinensis	Alkaloids L-ascorbic acid, anthocyanin, beta-carotene, beta-sitosterol	Boil in water and drink.	Hypotension, headache, stomach upset
6	Cumin	Cuminum cyminum	Essential oil	Powder added to food. Boil with water and drink on empty stomach.	-
7	Lemon	Citrus limon	Citric acid, ascorbic acid	Add to food. Drink the juice on empty stomach.	Gastric hyperacidity, tooth decay
8	Parsley leaves	Petroselinum crispum	Essential oil	Add to food. Drink the juice on empty stomach.	Headache
9	Aloe	Aloe vera	Vitamins and enzymes, saponins	Add to food. Drink the juice on empty stomach.	Hypoglycemia, skin rash, stomach pain, and cramps
10	Coconut oil	Cocos nucifera	Lauric acid, capric acid, caprylic acid	One tablespoon of the oil in empty stomach.	Headache, dizziness, skin rash
11	Grape fruits	Citrus paradisi	Flavones and isoflavones, anthocyanidin	Eat the fruit. Drink the juice.	Stomach upset, diarrhea
12	Senna	Senna alexandrina	Sennosides	Add to food.	Abdominal pain and cramps, nausea and diarrhea

In parallel, data about traditional mixtures are summarized in Table 2. The widely used herbs in weight-loss mixtures were ginger (eight times, 53.33%) and cinnamon (six times, 40%) as well as lemon, garlic, nettle, Senna, dandelion, ginseng, clove, and green tea with two uses for each (13.33%). Majority of these ingredients were boiled in water (11 mixtures, 73.33%). Out of the 15 mixtures, 12 mixtures (80%) were taken orally, while three mixtures (20%) were applied topically on the desired organ and rubbed. In addition, 12 mixtures (80%) were reported to have side effects. The major side effects in the oral mixtures were stomach upset, nausea, vomiting, diarrhea, and headache. On the other side, the major side effects in topically applied mixtures were skin irritation and rashes.

**Table 2:** Traditional herbal remedy mixtures used in weight loss.

S. No.	Composition	Form	Method of preparation	Method of use	Reported side effects
1	Lemons–Garlic	Solution in water	Mix the ingredients, and then boil.	Oral: Drink three small cups daily.	Decay tooth enamel, mouth sores, heartburn, burning sensation in mouth or throat
2	Ginger–Cinnamon	Solution in water	Mix the ingredients, and then boil.	Oral: Drink a cup before food.	Increased bleeding tendency, mouth sores
3	Olive oil–Ginger	Solution	Boil the oil in a pot, and add the ginger.	Topical: Apply on desired body part and rub.	-
4	Rose water–Apple cider vinegar–Bitter almond oil–Ginger	Solution	Mix the ingredients.	Topical: Apply on desired body part and rub.	Skin irritation and rashes
5	Ginger–Cumin–Cinnamon	Solution in water	Mix the ingredients, and then add boiled water.	Oral: Drink a small cup before food.	Hypoglycemia, breathing problems, bowel spasms
6	Nettle leaf–Eleuthero root–Senna leaf–Dandelion leaf–Marshmallow root–Slippery elm bark–papaya leaf–Sweet Cinnamon Bark–Orange peel–ginger root–Fennel seeds	Solution in water	Mix the ingredients, and then add boiled water.	Oral: Drink a small cup daily.	Mild stomach upset, fluid retention, drowsiness, skin irritation
7	Lemon balm–Chamomile flowers–Nettle eaf–Siberian ginseng root–St. John’s wort–Oats	Solution in water	Mix the ingredients, and then add boiled water.	Oral: Drink a small cup daily.	Severe allergic reaction, fluid retention, tiredness, restlessness
8	Ginseng extract–Lime juice–Cinnamon	Solution in water	Mix the ingredients, and then boil water.	Oral: Drink a small cup daily.	Insomnia, breast pain, tachycardia
9	Cinnamon–Ginger–Cardamom–Chicory–Black pepper–Star Anise–Cloves	Solution in water	Mix the ingredients, and then boil water.	Oral: Drink a small cup daily.	Swollen eyelids, shortness of breath, chest pain
10	Hibiscus–Ginger root–Dandelion leaf	Solution in water	Mix the ingredients, and then boil.	Oral: Drink a small cup daily.	Temporary stomach upset or pain, increased bleeding tendency
11	Masoor dal–Tomatoes–Garlic–Green chilies–Nigella seeds–Turmeric powder–Coriander leaves	Cooked in water	Cook all ingredients in a pressure cooker with water till tomatoes become softer.	Oral: Eat a dish.	Allergic rashes
12	Jasmine oil–Clove oil	Oil	Mix well.	Apply on desired organ with rubbing using circular motion for 10 minutes.	-
13	Bitter orange–Green tea–Senna	Solution	Shake all ingredients well.	Oral: Drink a small cup 30 minutes before breakfast and before lunch – to be taken five consecutive days and skip for two days.	Itching or redness of the skin, diarrhea
14	Green coffee bean–Green tea leaves	Solution in water	Mix the ingredients, and then boil.	Oral: Drink a cup 30 minutes before each meal.	Insomnia
15	Skimmed yogurt–Black tea–Cinnamon–Ginger–Curry leaves	Solution in water	Mix the ingredients, and then boil.	Oral: Drink a small cup daily.	-

Home remedies used for weight reduction

Data about home remedies are summarized in Table 3. The home remedies were either taken before meal or added directly to the meal. In addition, most reported side effects were gastrointestinal symptoms such as nausea, vomiting, and diarrhea as well as skin rash.

**Table 3:** Home remedies used in weight loss.

S. No	Common name	Major active constituents	Method of use	Reported side effects
1	Cedar vinegar	Pectin water-soluble vitamins	Add to food. Add to warm water and drink.	Tooth erosion, throat burn, gastric upset
2	Balsamic vinegar	Organic acids	To be rubbed on the desired organ.	Skin rashes
3	Yogurt	Bacterial cultures, lactose, and calcium	Eat one cup before meals.	Stomach upset, diarrhea
4	Fish oil	Omega-3 fatty acids	Add to food. Drink one tablespoonful before meal.	Bad breath, nausea, heartburn, skin rashes
5	Salmon	Protein omega-3 fatty acids, vitamins	Eat sautéed salmon.	Gastric upset, skin rashes
6	Honey	Fructose, glucose, and sucrose	Eat one tablespoonful before meal.	Nausea, vomiting
7	Gum	Arabinose, xylitol, galactose, and uronic acid	Continuous chewing all the day to reduce eating behavior.	Jaw pain, tooth decay, diarrhea
8	Water		Continuous drinking of water all the day to reduce eating behavior.	Frequent trips to bathroom

## Discussion

Obesity is not only confined to the developed world but has also extended toward the developing world [2]. Billions of dollars are spent for weight-loss products every year around the world. It is now estimated to reach 60 billion dollars in United States alone. Yet each year, millions of people are still dying of obesity or its related diseases. Obesity has now become one of the major health concerns all over the world [1].

Saudi Arabia has become greatly westernized, over past few decades, and now it has been considered to have one of the highest rates of incidence of obesity and overweight [5]. Obesity is a major concern in Saudi Arabia, where seven out of 10 people are experiencing this problem. Previous obesity prevalence studies related to Saudi Arabia indicated wide spread of obesity and overweight, enhancing prevalence of other major diseases such as hypertension, diabetes, hyperlipidemia, obstructive sleep apnea, and osteoarthritis representing economic burden. In addition, consumers did not find a trained person for getting accurate information about herbal remedies. A previous study in Saudi Arabia illustrated that only 36% of pharmacists provide patients with counseling regarding the use of herbal medicine [9].

Some of the remedies used in Tabuk area were previously approved for their efficacy in reducing weight such as ginger [10], cinnamon [11], green tea [12], coffee beans [13], chili [14], hibiscus [15], curcumin [16], kidney beans [17], and cumin [18]. We want to add that most of the studies on weight reduction remedies were performed only on animals, and very little studies were performed on humans. Many of these clinical studies gave controversial results such as those conducted on fish oil [19], dairy products [20], fibers [21], and aloe [22]. The weight reduction effects of other remedies, that are widely used, were not studied previously such as parsley, grape fruit, rose water, apple cider vinegar, bitter almond, nettle leaf, eleuthero root, dandelion leaf, marshmallow root, slippery elm bark, papaya leaf, balsamic vinegar, and bitter orange. In addition, some of the remedies were reported to have severe side effects as coconut [23], senna [24], and bitter orange [25]. Finally, it is important to highlight that herbal and home remedies differ widely from one place to another depending on society culture, endogenous weather, and available medicinal plants.

The widely used medicinal plant for weight reduction was ginger (*Zingiber officinale* Roscoe, family: Zingiberaceae). It contains volatile oils (1% to 3%) including terpenoids such as zingiberene and pungent components such as gingerol, shogaol, and zingerone. Ginger produced weight reduction effects through several mechanisms such as increasing thermogenesis, enhancing lipolysis, suppressing lipogenesis, suppressing appetite, and blocking fat absorption [10]. Two clinical trials were conducted to evaluate the weight reduction effects of ginger, and they revealed minor beneficial effects [26,27].

Another widely used medicinal plant for weight reduction is cinnamon (*Cinnamomum zeylanicum*). The main components of cinnamon are cinnamic acid, cinnamaldehyde, eugenol, and coumarin. It is reported to enhance insulin sensitivity and cellular glucose uptake as well as produce anti-inflammatory and antioxidant effects [11]. Clinical trials on cinnamon lead to controversial and inconsistent results concerning its weight reduction effects. Some of the studies revealed beneficial effects of cinnamon on body composition, while others did not support this effect [28].

One of the most widely used herbs for weight loss is green tea. It is marketed in various forms: green tea prepared in an unoxidized form, partially oxidized, and black tea undergoing complete oxidation process. All forms of teas contain high amounts of catechins and gallic acid [29]. It has been reported previously that green tea possesses the ability to inhibit adipogenesis by enhancing energy expenditure through different mechanisms such as thermogenesis, oxidation of fat, and excretion of lipid. Consistently, black tea has greatly improved obesity-associated complications by attenuating visceral fat deposition and decreasing hepatic triglyceride levels [12].

Green coffee is another commonly used beverage. Coffee is a complex mixture of chemical components, which is greatly affected by coffee bean species (*Coffea canephora* and *Coffea arabica*), the brewing and the roasting processes. The chemical composition of green coffee beans before roasting is 6.5%-10% chlorogenic acid (CA), 1.2%-2.2% caffeine, 10%-16% lipids with special diterpenes (cafestol and kahweol), 45%-52% carbohydrate, 0.7%-1.0% trigonelline, 11% protein, and 4.2%-4.4% minerals. Chlorogenic acid represented weight-loss properties of coffee [13].

Organic acids were the main bioactive compounds that are responsible for therapeutic effects of *Hibiscus sabdariffa* besides other compounds such as flavonoids, anthocyanins, and phenolic acids. Different studies have attributed the ability of Hibiscus extract to fight obesity and correlate it with one or more specific compounds in its composition [30]. For example, the ability of hibiscus to reduce weight is attributed to its contents of anthocyanin, polyphenol, or organic acids such as hibiscus, dimethyl hibiscus, and hydroxycitric acids [15].

Curcumin is a naturally occurring member of ginger family (Zingiberaceae) and considered one of the widely investigated medicinal plants. It is noted for possessing anti-obesity effects. A randomized controlled study evaluated the efficacy of 30 days consumption of curcumin in weight reduction of overweight human subjects associated with lifestyle intervention. Curcumin enhanced weight loss from 1.9% to 4.9% and helped reduction of fat mass, as well as waist and hip size. It is reported to activate brown adipose tissue [16].

Regarding home remedies, many people used different materials especially fish oil, which is rich in omega-3 polyunsaturated fatty acids, such as eicosapentaenoic acid or docosahexaenoic acid that have reported to produce cardioprotective, anti-inflammatory, and hypotriglyceridemic effects. However, these fatty acids may reduce obesity comorbidities, especially by improving metabolic syndrome. Regarding supplementation with polyunsaturated fatty acids alone, studies report no change in body weight, but it assesses weight loss using dietary modification and physical activity [19].

Another home remedy used for overcoming obesity is yogurt. Yogurt and milk have generally similar nutrient composition, yogurt is a relatively unique dairy product because during manufacturing procedures and fermentation, many nutrients such as protein, riboflavin, vitamin B-6, vitamin B-12, calcium, potassium, zinc, and magnesium are highly concentrated in yogurt than milk. This increase is ranging from 20% to more than 100% in yogurt than in milk. A recent meta-analysis summarizes the results from 29 researches that examined effects of various dairy interventions. They reported that dairy interventions produced no significant weight loss but only produced a modest reduction in fat mass. It is only useful when used as part of an energy-restricted diet. Dairy interventions did not promote more loss in weight or fat mass than did control dietary regimens among trials with *ad libitum* interventions [20].

Many people used complex fiber supplements to reduce their weight. A systematic review investigated the effect of fiber supplements as a complementary treatment for weight management. They found that fiber supplements without weight-loss interventions did not produce considerable anti-obesity effects. One of the widely used products is gum Arabic, dextrin- and pectin-rich fiber with no dietary interventions, which indicated a reduction in body weight and energy intake [21].

Although, there is no clear documentation about efficacy of herbal and home remedies, their use has increased dramatically worldwide especially in the field of weight reduction. Therefore, conducting comprehensive studies about herbal and home remedies that reduce weight is essential. It is important to investigate their bioavailability, effectiveness, invasiveness, side effects, toxicities, body toleration, and ethical obligation. In addition, we should evaluate any available data about clinical trials that deal with weight reduction herbal medicine. Considering these relevant issues helps physician and other healthcare providers to deal easily with their patients, solve their problems, and educate them about these therapies. Finally, results of studies and clinical trials will help governmental control of herbal medicine and enhance implementation of local regulations for safety and efficacy of herbal products. Although some herbal remedies are available in pharmaceutical dosage forms, most of these products are purchased from herbal shops in their crude form without any evidence about their safety and purity.

## Conclusions

Many natural herbal and home remedies are still in use in Tabuk. These products are used for many purposes especially for weight loss. However, many of the herbal or home remedies used for weight loss may lack important scientific evidence for their usage, effectiveness, or safety. In addition, many of the used mixtures of herbal medicine for weight loss do not have previous reports about their interactions with each others or their interactions with other foods and drugs. 
